# 3D mapping of native extracellular matrix reveals cellular responses to the microenvironment

**DOI:** 10.1016/j.yjsbx.2018.100002

**Published:** 2018-12-19

**Authors:** Zipora Lansky, Yael Mutsafi, Lothar Houben, Tal Ilani, Gad Armony, Sharon G. Wolf, Deborah Fass

**Affiliations:** a Department of Structural Biology, Weizmann Institute of Science, Rehovot, Israel; b Department of Chemical Research Support, Weizmann Institute of Science, Rehovot, Israel

**Keywords:** Extracellular matrix, Cryo electron microscopy, Collagen VI, Fibronectin, Tomography, Scanning transmission electron microscopy

## Abstract

Cells and extracellular matrix (ECM) are mutually interdependent: cells guide self-assembly of ECM precursors, and the resulting ECM architecture supports and instructs cells. Though bidirectional signaling between ECM and cells is fundamental to cell biology, it is challenging to gain high-resolution structural information on cellular responses to the matrix microenvironment. Here we used cryo-scanning transmission electron tomography (CSTET) to reveal the nanometer- to micron-scale organization of major fibroblast ECM components in a native-like context, while simultaneously visualizing internal cell ultrastructure including organelles and cytoskeleton. In addition to extending current models for collagen VI fibril organization, three-dimensional views of thick cell regions and surrounding matrix showed how ECM networks impact the structures and dynamics of intracellular organelles and how cells remodel ECM. Collagen VI and fibronectin were seen to distribute in fundamentally different ways in the cell microenvironment and perform distinct roles in supporting and interacting with cells. This work demonstrates that CSTET provides a new perspective for the study of ECM in cell biology, highlighting labeled extracellular elements against a backdrop of unlabeled but morphologically identifiable cellular features with nanometer resolution detail.

## 1 Introduction

The extracellular matrix is a complex and dynamic network of interacting fibrils and associated factors that exists in intimate communication with cells. Cells produce and secrete ECM precursor proteins, sense and respond to ECM structure, composition, and mechanical properties, and re-uptake and digest ECM for turnover and nutrients (Lu et al., 2012). The composition, modifications, and physical and functional properties of the ECM vary according to tissue type (Naba et al., 2016) and health vs. disease (Iozzo and Gubbiotti, 2018). For example, variations in ECM properties are associated with cancer invasiveness (Chang et al., 2017) and aging (De Luca, 2018). Furthermore, molecular defects in ECM proteins can impact cell activities. A case in point is the relationship of collagen VI mutations to mitochondrial function in muscular dystrophies (Irwin et al., 2003, Zamurs et al., 2015). Appreciating the factors that influence tissue homeostasis or its breakdown requires a deep understanding of the interdependence of cells and ECM.

Due to the importance of ECM ultrastructure and ECM-cell interactions, imaging techniques have been instrumental in both raising and answering questions related to ECM organization and function. Transmission electron microscopy (TEM) provided early evidence of the connection between the cytoskeleton and the ECM (Singer, 1979), leading to the discovery of integrin-mediated cell-ECM contacts (Wolfenson et al., 2013). TEM was also used to discover the banding patterns of collagens (Gross and Schmitt, 1948, Hodge and Schmitt, 1959, Hulmes et al., 1981, Schwartz et al., 1969), whereas scanning electron microscopy (SEM) has provided information on collagen organization in tissues (Gillies et al., 2014, Young et al., 2014). These techniques have largely relied on isolation of ECM or tissue fixation and embedding.

Here we visualize the native arrangements of ECM components in a fully hydrated state and in association with the cells that produced them, while identifying relationships between organelles, cytoskeleton, and ECM patterning, using state-of-the-art electron microscopy and sample preparation methods. The delicate structural organization of many ECM fibrils makes them sensitive to perturbation during purification or other sample processing procedures, motivating structural studies *in situ*. The technique of scanning transmission electron microscopy (STEM) has historically been used in materials science but was recently adapted for cryo-preserved biological samples (Wolf et al., 2017, Wolf et al., 2014). CSTET allows for reconstructions at low-nanometer resolution of cell regions up to or even exceeding 1 μm in depth, with contrast that reflects atomic composition (Rez et al., 2016). Sample preparation for CSTET consists of rapid vitrification of live cells, with no fixation, dehydration, coating, or sectioning required. Cryo-preservation thus reduces the likelihood of structural deformations and staining artifacts, which can occur in traditional sample preparation methods (Chan and Inoue, 1994). Although most ECM components have low contrast with poor direct visibility, a detailed read-out of their locations and patterns in relation to cells can be obtained by adding gold-conjugated antibodies shortly before plunge-freezing. The STEM imaging modality is an advantage in this regard, as it allows for precise localization of small gold labels due to their high contrast.

In this report, we used CSTET to investigate the three-dimensional organization of collagen VI and fibronectin secreted by and in association with cultured fibroblasts. Along with comprehensive maps of these ECM networks, the contours and interior contents of cells were simultaneously visualized, providing morphological details of cellular organelles such as vesicles and mitochondria. Cryo-preserved samples provided snapshots of dynamic cellular and subcellular processes such as cell spreading and retraction, mitochondrial fission, ECM remodeling, and ECM recycling. Using CSTET, we elucidated how ECM organization influences organelle morphology and cytoskeletal distribution in cells growing in a topographically heterogeneous environment. The interplay between micron-scale variations in the architecture of the cell support and the deposition of ECM was observed to tune cell structure and dynamics.

## 2 Materials and methods

### 2.1 Cell culture and CSTET sample preparation

WI-38 lung fibroblasts were purchased from Coriell and maintained in Minimal Essential Medium (MEM) supplemented with 15% fetal bovine serum, L-glutamine, and antibiotics as recommended by the supplier. For CSTET, cells were grown on electron microscopy (EM) grids (Quantifoil R 3/5 or 0.6/1, 200 mesh, gold) by placing the grids onto a tissue culture coverslip affixed using a ring of warmed parafilm to the bottom of a 3.5 cm tissue culture dish. Cells were seeded onto the coverslip and grids, and cultures were grown for 5–7 days to near confluence before immunogold labeling.

For labeling, fibroblast-covered grids were incubated typically for 1 hr at 37 °C in a tissue culture incubator with primary antibody against either collagen VI (Abcam ab6588, rabbit against full-length purified human collagen VI) or fibronectin (Dako A 0245, rabbit against full-length purified human fibronectin) diluted 1:200 in MEM. After incubation, grids were washed in phosphate buffered saline (PBS) by passing each grid through five 40-μl drops of PBS, for 1–2 s in each drop. The grids were then incubated for 45–60 min at 37 °C in a tissue culture incubator with colloidal gold-conjugated secondary antibody (EM grade goat-anti-rabbit IgG, 6 nm gold) diluted 1:20 in MEM. Grids were washed again in PBS just before vitrification, which was performed by rapid plunge-freezing in liquid ethane using a Leica EM-GP plunger. Prior to plunging, grids were blotted with filter paper at 93% humidity and 22 °C for 3 sec from the side opposite the cells. Grids were stored in liquid nitrogen until use.

### 2.2 Scanning electron microscopy

For SEM, cells were grown on glass coverslips in 24-well tissue culture dishes, washed briefly with PBS containing calcium and magnesium (PBS++), and fixed with freshly prepared paraformaldehyde (4%) and glutaraldehyde (2.5%) in PBS for 1 h. Samples were then washed twice with PBS and twice with water, followed by dehydration through an ethanol series beginning with 30% and changing to solutions of 50%, 70%, 90%, and three times 100%, over 30 min. They were then dried in a critical point dryer. To preserve the three-dimensionality and fine structure of the ECM, no heavy-metal coating was applied, and care was taken to sputter coat with only a very fine layer of carbon, sufficient to minimize charging effects in sample visualization. Samples were imaged in an Ultra microscope (Zeiss).

### 2.3 CSTET data acquisition

CSTET data were collected as previously described (Wolf et al., 2017, Wolf et al., 2014) using a Tecnai F20 electron microscope (Thermo Fisher Scientific) with a 200 kV Schottky field-emission gun. Extraction voltage was set to 4300 V, gun lens 6, condenser aperture 10 μm, camera length 320 mm, and spot size 5, with probe diameter of approximately 2 nm and a semi-convergence angle of approximately 1 mrad. Vitrified samples were loaded into Gatan 626 or 914 cryo-holders, and STEM images were collected with both a bottom-mounted bright-field detector (Gatan model 807) and a Fischione high-angle annular dark-field detector located at the 35 mm port of the microscope column. 4096 × 4096 images were recorded with 0.5 µsec pixel dwell time. Spatial sampling was set to 1.2 nm/pixel. Tomograms were collected (using Serial EM software) by tilting the sample along a single axis in 2° increments, from −20° to 60°, and then from −22° to −60°.

### 2.4 Tomogram reconstruction

IMOD software was used for all steps of tomographic reconstruction (Kremer et al., 1996). Fiducial alignment of tilt series was done using the antibody-conjugated gold beads as markers. Aligned stacks were binned by 3 when tomograms were used for visual representation and binned by 2 when used for collagen VI bead analyses. Tomographic reconstruction was done by weighted back-projection. For visual clarity, tomograms were filtered with non-linear anisotropic diffusion using Amira 6 software (Thermo Fisher Scientific).

### 2.5 Segmentation for visualization

For visual representation, tomograms were segmented and rendered using Amira 6. Cellular organelles, fibronectin, and plasma membrane were segmented manually using the brush feature. Microtubules were segmented and visualized using filament tracer. Segmentation for gold beads was done by setting an intensity threshold. Visualization for cellular organelles and fibronectin was done by volume rendering. Plasma membrane was visualized by surface generation. Gold beads were visualized by using material statistics to find centers of mass of segmented beads, exporting the spreadsheet to point cloud, and representing the point cloud with spheres.

### 2.6 Collagen VI bead analyses

To counteract segmentation artifacts arising from the “missing wedge” (Leis, 2018), the centers of mass of the gold beads were further refined. The refinement decreases the likelihood that the point-spread image of a single gold bead associated with the missing wedge becomes segmented into multiple centers of mass. In the first step, peak finding algorithms (Crocker and Grier, 1996) were applied to isolate gold beads in two dimensional slices of the tomogram. In the second step, nearest-neighbor peaks closer than the size of the gold bead in successive slices along the direction of the missing wedge were merged using custom-written software. For distance analyses of peaks, a pair correlation function was written in MATLAB according to the equations presented in Peckys et al. (Peckys et al., 2015). The function was written for analyses of particles in two dimensions and contained a covariance function for edge correction and a smoothing kernel with the bandwidth set to 5.

### 2.7 MS proteomics

Confluent WI-38 fibroblasts grown on tissue culture plates were washed with cold PBS++, and cells were removed by 5 min incubation in ECM enrichment buffer (Vlodavsky et al., 1987) supplemented with complete protease inhibitor mixture (Sigma Aldrich), followed by washing with PBS++. Enriched ECM was then either directly processed for MS or first deglycosylated, and results from both approaches were pooled for presentation in Table 1. For directly processed samples, ECM was scraped into 1.5 ml tubes, and excess buffer was removed by centrifugation. The ECM was then sonicated in 6 M urea, 50 mM Tris buffer, pH 8.0, followed by digestion with trypsin/LysC protease mixture (Promega). Digestion was stopped with 0.5% TFA. After titration to neutral pH, peptides were reduced with 5 mM dithiothreitol, alkylated with 10 mM iodoacetamide, desalted using solid-phase extraction columns (Oasis HLB, Waters), and dried by rotary evaporation. To prepare deglycosylated samples, enriched ECM was reduced and alkylated on the culture plate in 20 mM Tris, pH 8, 6 M guanidine HCl. Samples were then deglycosylated using either deglycosylation mix II (New England BioLabs) or trifluoromethanesulfonic acid. Deglycosylated samples were scraped from the plates and briefly run into a polyacrylamide gel, from which they were digested with trypsin.

**Table 1:** Predominant ECM proteins in WI-38 cultures identified by MS.

Protein	UniProt accession	sequence coverage	# validated peptides	# validated spectra
fibronectin	P02751	86%	900	6606
collagen VI α-3	P12111	75%	519	3351
heparan sulfate proteoglycan	P98160	58%	318	1473
fibrillin-1	P35555	62%	308	1123
tenascin	P24821	67%	190	910
fibrillin-2	P35556	61%	256	904
collagen VI α-1	P12109	61%	120	775
collagen VI α-2	P12110	57%	140	715
latent-transforming growth factor β-binding protein 1	Q14766	54%	148	641
transforming growth factor-beta-induced protein ig-h3	Q15582	69%	88	633

ECM peptides were separated by nano-flow reversed phase chromatography coupled to a Fusion Lumos mass spectrometer (Thermo Scientific). MS1 resolution was 120,000 and MS2 resolution was set to 15,000. MS/MS spectra were identified using SearchGUI version 3.2.20 (Vaudel et al., 2011) with the search engines X!Tandem (Craig and Beavis, 2004), Andromeda (Cox et al., 2011), and MyriMatch (Tabb et al., 2007). The search database was a concatenated target/decoy (Elias and Gygi, 2010) version of the SwissProt human database (downloaded on 27.12.2016, 20283 target sequences) (Apweiler et al., 2004). Peptides and proteins were inferred using PeptideShaker version 1.16.12 (Vaudel et al., 2015). Peptide Spectrum Matches, peptides, and proteins were validated at a 1.0% false discovery rate estimated using the decoy hit distribution.

The mass spectrometry data along with the identification results were deposited in ProteomeXchange Consortium (Vizcaíno et al., 2014) via PRIDE partner repository (Martens et al., 2005). Dataset identifier is PXD007700.

## 3 Results

### 3.1 Composition of fibroblast ECM

**Fig. 1:**
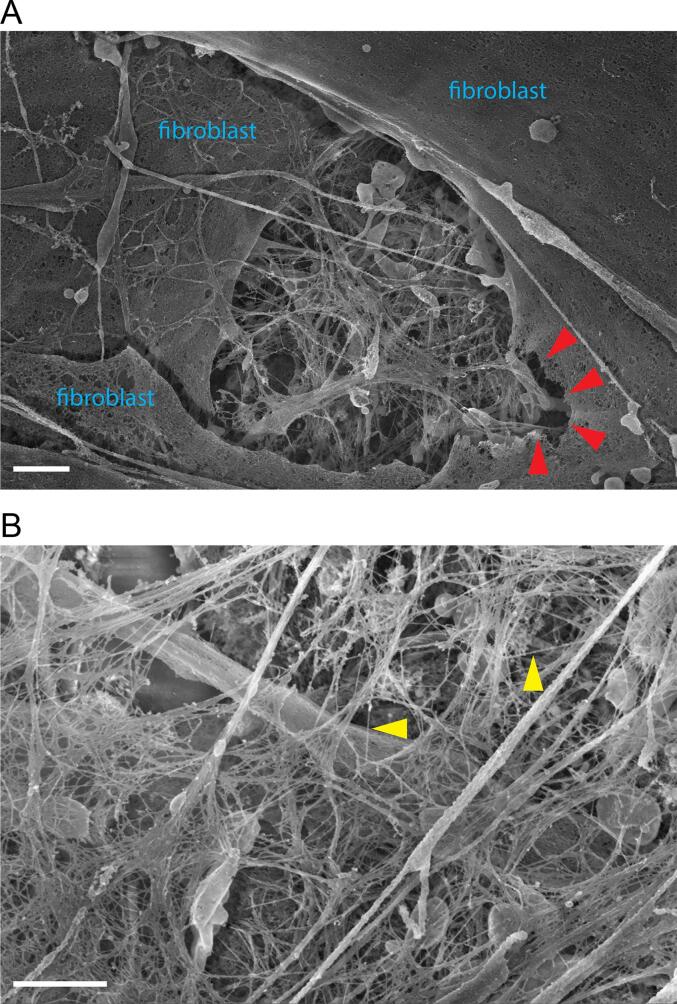
SEM imaging of fibroblast ECM. Scale bars are 1 μm. (A) ECM deposited by confluent cultured fibroblasts is visible in the center of this image, but damage to the cell membrane is apparent (red arrowheads). (B) ECM fine structure, including suspended microfilaments ∼20 nm in thickness (yellow arrowheads). (For interpretation of the references to colour in this figure legend, the reader is referred to the web version of this article.)

The data presented herein were collected using primary WI-38 embryonic lung fibroblasts. Prior to the CSTET study, we performed two experiments to analyze the structure and composition of ECM from these cells: SEM and mass spectrometry (MS) proteomics. SEM micrographs of confluent fibroblast cultures (Fig. 1A) revealed a meshwork of proteins with a variety of fibril thicknesses and appearances, giving preliminary insight into ECM network patterns (Fig. 1B). However, due to the requirements for SEM sample preparation, including dehydration and surface coating with carbon or other elements (see Materials and methods), the fine structure and three-dimensionality of the ECM were rarely preserved with the high quality of the image presented. Furthermore, identifying individual fibers was challenging, since we found immunogold labeling in SEM to be less robust and reproducible than in immunofluorescence microscopy (not shown). MS analyses revealed the predominant ECM proteins in these cultures to be fibronectin, collagen VI, fibrillin, and tenascin (Table 1).

### 3.2 CSTET imaging of ECM in association with fibroblast cells

**Fig. 2:**
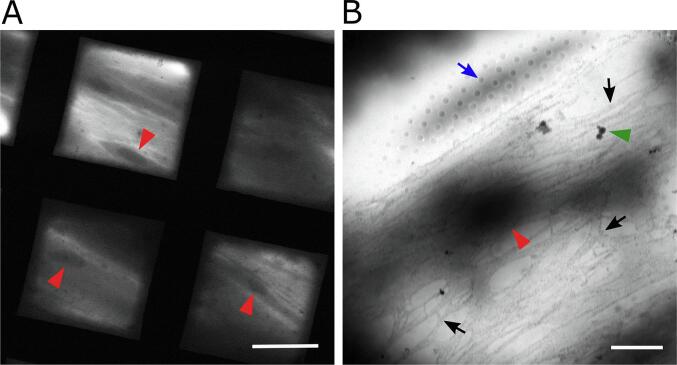
STEM imaging of fibroblasts at low magnification. (A) Fibroblasts seen through squares of an EM grid. Nuclei indicated by red arrowheads. Scale bar is 50 μm. (B) Single grid square showing a fibroblast cell spread across the carbon support. The blue arrow points to a hole (0.6 µm diameter) in the support. The green arrow indicates ice contamination. The nucleus is indicated by a red arrowhead. Fibronectin fibers labeled with 20 nm gold beads are visible as a gray network covering the cell (select fibers are indicated by black arrows; individual gold beads are not visible on this scale). Scale bar is 5 μm. (For interpretation of the references to colour in this figure legend, the reader is referred to the web version of this article.)

Based on the above observations, fibroblast cultures were prepared for CSTET analysis. Collagen VI and fibronectin were chosen for study due to their abundance and robust labeling with gold-conjugated antibodies. Furthermore, collagen VI has a well-known beaded microfibril pattern (Baldock et al., 2003, Bruns, 1984, Engel et al., 1985, Engvall et al., 1986), which provided a frame of reference. Fibroblasts were grown on gold Quantifoil EM grids for five to seven days until cells reached approximately 80–90% confluence (Fig. 2A), enough to allow the shift of cellular energy resources from proliferation to ECM production (Lemons et al., 2010), but not fully confluent so as to prevent imaging due to excessive sample thickness. The fibroblasts attained an elongated, bipolar morphology (Fig. 2A), similar to fibroblasts *in vivo*. After short incubations with primary antibodies followed by gold-labeled secondary antibodies, cells and associated ECM were preserved by rapid vitrification. Areas of the cultures rich in ECM could be visualized by light microscopy under cryogenic conditions for correlation purposes prior to cryo-STEM microscopy by using a mixture of gold- and fluorescence-labeled secondary antibodies (not shown). However, fibroblast cultures were sufficiently rich in ECM (Fig. 2B) that the CSTET results presented herein could be obtained without correlative mapping. For electron microscopy studies of cells, grids with perforated carbon coatings containing holes of defined sizes and spacings are conventionally used (Fig. 2B). In addition to facilitating the vitrification process (see Methods), the holes provided variation in the cell-growth substrate, which we used to investigate how ECM deposition and cell morphology respond to micron-scale heterogeneity in surface rigidity.

For CSTET data collection, scanning was performed with a spatial sampling of 1.2 nm per pixel. Binning during data processing produced tomograms with a sampling of 3.5 nm per pixel. Though this resolution is lower than attainable by cryo-TEM, thicker regions of cells and associated ECM could be examined by CSTET (Rez et al., 2016).

### 3.3 CSTET of gold-labeled fibronectin highlights its mechanical support role

**Fig. 3:**
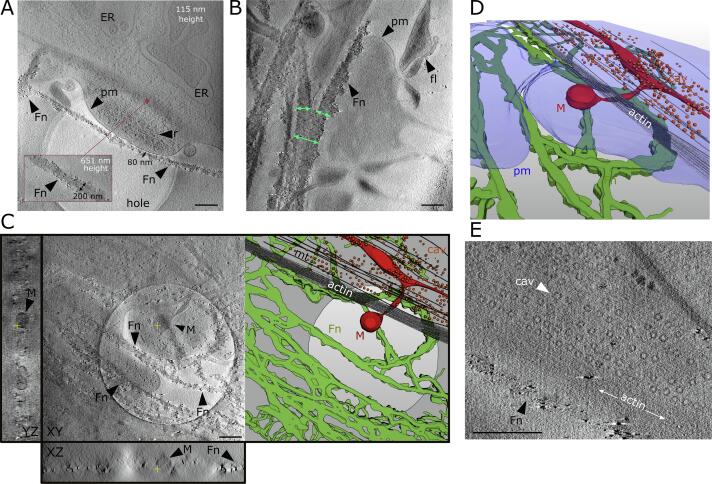
CSTET imaging of cells and gold-labeled fibronectin. Scale bars are 500 nm. Labeled cell features are endoplasmic reticulum (ER), plasma membrane (pm), filopodia (fl), mitochondria (M), caveolae (cav), microtubules (mt), ribosomes (r), and actin. (A) A slice from a CSTET reconstruction shows gold-labeled fibronectin (Fn) along the edge of a fibroblast. A thin (∼80 nm diameter) fibronectin bundle is labeled. A thick (∼200 nm) bundle, at a different height in the tomogram, is shown in the inset. Height is measured from the carbon support, identified as the slice where the edges of the hole appear the sharpest and where the carbon area appears grainy. (B) Fibronectin bundles show non-uniform thickness and rough surfaces. Green arrows indicate the widths of fibronectin fibers to either side of a branch-point. (C) Left panel shows a z-slice of a tomogram with plentiful fibronectin cables spanning a hole in the carbon support. The hole and the fibronectin can be simultaneously seen in the slice shown due to suboptimal resolution in z. The mitochondrion is centered at a different depth in the tomogram but can also be detected faintly in this slice. Orthogonal slices are shown in rectangular boxes besides the z-slice, with yellow crosses indicating the volume from which they were taken in other slices. The right panel shows segmentation of the fibronectin fibers (green), carbon (light gray), mitochondria (red), caveolae (orange), and cytoskeleton (dark gray) from the entire tomographic reconstruction. (D) Tilting the segmentation of the tomogram reveals the three-dimensional relationship between mitochondria and cytoskeleton, and the additional segmentation of the cell surface shows how plasma membrane and intracellular organelles are affected by the presence of a hole but cytoskeleton and ECM remain extended across the hole. (E) Close-up view reveals the parallel arrangement of actin, fibronectin, and rows of caveolae. (For interpretation of the references to colour in this figure legend, the reader is referred to the web version of this article.)

Tomograms were collected of samples that were immunogold-labeled for fibronectin. The abundant labeling of fibers of various thicknesses was consistent with the known supramolecular assembly of fibronectin, in which thin fibrils cluster to form thicker bundles (Singh et al., 2010) (Fig. 3A). The finest structures observed were less than 50 nm thick, and the largest bundles were 600 nm thick. Gold labels were seen to decorate the outsides of the bundles but not to penetrate their interiors (Fig. 3A, B). Thick fibrils aligned roughly with the long cell axis and cytoskeletal elements (Fig. 2B and Fig. 3A). Fibronectin bundles tended to show bulges and irregularities (Fig. 3B) in agreement with previous reports (Chen et al., 1997, Peters et al., 1998), and bundle branch-points resembled those observed by high resolution light microscopy (Früh et al., 2015), with conservation of total filament width at the branch-point as for a ripping/splitting or a merging process (Fig. 3B). Despite width conservation upon branching, fibrils were sometimes seen to taper (Fig. 3B), suggesting that fibronectin maturation can occur by merging of protofibrils of different lengths.

The well-defined surfaces of the fibronectin bundles, delineated by gold labeling and density differences between the protein and the surrounding environment, enabled segmentation of the bundles. Cell surfaces, intracellular organelles, and cytoskeleton were also segmented and rendered (Fig. 3C). Cytoskeletal elements (actin filaments and microtubules) were seen to extend over holes in the carbon support with no apparent disruption. In contrast, holes influenced cell contours and the locations and shapes of membrane-bound organelles such as mitochondria (Fig. 3C and Movie S1). Tomograms clearly revealed the three dimensional relationship between cytoskeleton, organelles, and fibronectin (Fig. 3D). Specifically, a thick bundle of filamentous actin (200 nm in diameter) was observed to pass over the neck of a mitochondrial offshoot extending from a parent mitochondrion into a bleb-like region of the cell filling a hole in the carbon substrate (Fig. 3C, D). In the tomogram, the bulbous end of the mitochondrial offshoot, with a diameter of 420 nm, rests directly above a fibronectin cable spanning the hole. This cable is part of a basket-like network that stretches across the hole and partially supports the cell bleb. Another example of the spatial relationship between ECM and cellular features is the observation of fibronectin fibrils and actin cytoskeleton running parallel to rows of caveolae on the cell surface (Fig. 3E). In some tomograms, plasma membrane containing large numbers of caveolae lie directly over patches of fibronectin, which may reflect the proposed role of caveolae in fibronectin turnover (Sottile and Chandler, 2004, Thompson et al., 2017).

### 3.4 Branching and bundling of collagen VI in association with cells

**Fig. 4:**
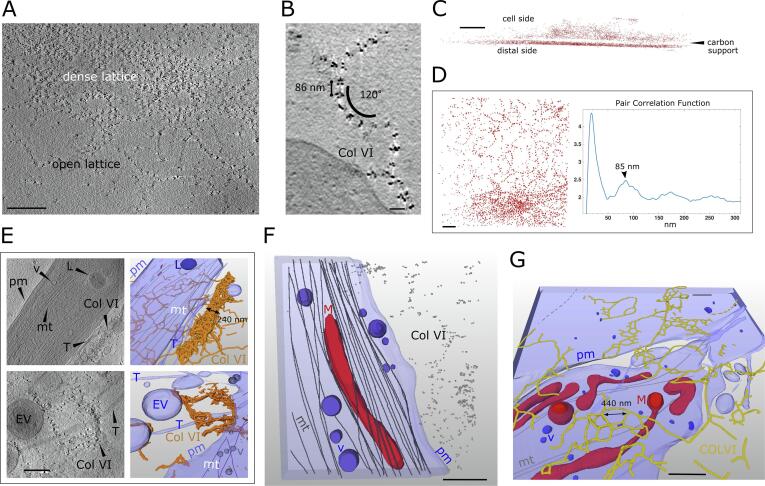
CSTET imaging of cells and gold-labeled collagen VI. Scale bars are 500 nm, except for panel B where it is 100 nm. Labeled cell and ECM features are plasma membrane (pm), extracellular vesicles (EV), membrane tendrils (T), collagen VI (Col VI), microtubules (mt), intracellular vesicles (v), and lysosome (L). (A) Collagen VI arranged in dense and open lattices. (B) Close-up view of a tomographic z slice showing the ∼85 nm periodicity of gold-bead labels, and curvature of a collagen VI filament. (C) Side view of gold-bead centroids from automated segmentation. (D, left) Top view of gold-bead centroid positions on the distal side of the carbon in a slice 200 nm thick. (D, right) Pair correlation analyses on the same bead positions displaying a peak at 85 nm, corresponding to the most probable distance between gold-bead clusters. (E) Thick collagen VI bundles observed in slices from tomograms (left) and segmentation of the same tomograms (right). (F) An open-lattice pattern of collagen VI gold labels (small gray spheres) adjacent to a cell displayed with segmented organelles and cytoskeletal elements. Here collagen VI labeling extends to the cell edge but is not seen under the cell. (G) Tilted view of a segmented tomogram, carbon side up. Much of the segmented collagen VI is between the cell and the carbon.

Tomograms were collected of cell cultures with immunogold labeling of collagen VI. Labeling was pervasive and revealed both open and dense lattices (Fig. 4A). In contrast to the bundles of fibronectin, which excluded gold-labeled antibodies from their interiors, bundles of collagen VI were permeable to label. The polyclonal antibody used for labeling was raised against the full-length protein, but the gold observed in the tomograms was clustered at regular intervals (Fig. 4B), recalling the beads-on-a-string appearance of collagen VI (Baldock et al., 2003, Bruns, 1984, Engel et al., 1985, Engvall et al., 1986, Godwin et al., 2017). As observed previously for collagen VI polyclonal antibodies (Kielty et al., 1991), the primary epitopes were likely concentrated in the globular domains that form the fibril bead regions. The most common distance between successive gold-bead clusters was measured to be about 85 nm (Fig. 4B).

**Supplementary Fig. S1:**
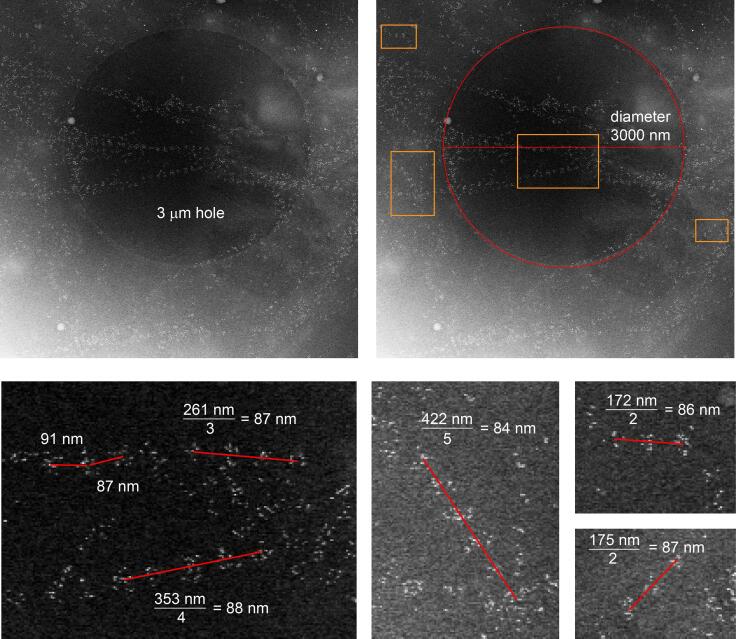
Direct measurement of collagen VI repeat distance. The upper panels show a cryo-STEM dark-field image of immunogold labeled collagen in the vicinity of a 3 µm (3000 nm) hole in the carbon coating of the EM grid. The bottom panels show magnified (5x) views of selected regions of the image with direct measurements of the repeat period.

To obtain a global measure of distances between collagen VI label clusters in the tomograms, but at the expense of information regarding the connectivity of the clusters along fibrils, gold distribution was analyzed using a pair correlation function (Peckys et al., 2015). As input for the analysis, the coordinates of the gold labels in a tomogram were determined by searching for local brightness maxima in areas corresponding to the known size of the gold beads (6 nm diameter). Remarkably, we observed extensive gold labeling of collagen VI on both sides of the carbon support (Fig. 4C), despite the fact that cells had been seeded on only one side. This observation demonstrates the ability of fibroblasts to disperse collagen VI widely in the cell environment, which may contribute to its non-cell-autonomous effects in tissues (Bönnemann, 2011). On the cell side, labeled collagen was more heterogeneously distributed over cells and exposed carbon (Fig. 4C), reaching thicknesses up to 500 nm in some tomograms. For the distance analysis, the nearly 8000 gold beads on the distal side of the carbon were used, since these were distributed flat against the carbon and thus could be analyzed in two dimensions (Fig. 4C, D left). The correlation analysis revealed a prominent peak at about 85 nm (Fig. 4D, right), corresponding to the typical distance between successive label clusters measured along the collagen VI fibrils (Fig. 4B). An additional peak centered at a shorter distance (18 nm) is due to the multiple gold labeling of the same collagen VI globular bead region. Notably, the 85 nm repeat distance measured by CSTET is shorter than values typically obtained from isolated collagen VI oligomers or fibril fragments examined by AFM or by electron microscopy techniques involving rotary shadowing or other methods of sample preparation (Baldock et al., 2003, Bruns, 1984, Engel et al., 1985, Koudouna et al., 2014). To validate the CSTET measurements, supplementary images are presented showing holes in the carbon grid as size references and direct measurements of the repeat distance of collagen VI gold-label clusters (Fig. S1).

As for fibronectin, tomograms of collagen VI-labeled samples were segmented and rendered (Fig. 4E-G). Contrast for collagen VI fibrils was not as strong as for fibronectin, but fibrils could readily be segmented by tracing successive gold-labeled beads, sometimes aided by the faint contrast of the fibrils between beads. With the caveat that cells may have limited the labeling of collagen VI deposited between the cell and the carbon support (Fig. 4F and Movie S2), the association of collagen VI with cells and its distribution around cells could be mapped. The top panels of Fig. 4E show an elongated cell flanked on one side by a thick and wide (200 nm), three-dimensional collagen VI bundle. Elsewhere in the cell vicinity, open collagen VI lattices are seen spread against the carbon support. The bottom panels of Fig. 4E show dense three-dimensional labeling of collagen VI interspersed with bulbous cell projections and membranous extensions that may be retraction fibers.

Commonly observed spanning multiple cells was an extended, irregular network of collagen VI (Fig. 4G). Analysis of this network revealed nodes with typically three, or occasionally four, branches. Clusters of networked collagen VI formed polygons a few hundred nanometers across (Fig. 4G), and these clusters were interspersed with extended, uninterrupted single fibrils with lengths up to about 1 μm. Collagen VI was sometimes located at cell-cell contact zones, similarly to a previous report of collagen VI bridging chondrocytes (Godwin et al., 2017). Single collagen VI fibrils were also seen to line cell edges for distances exceeding 4 μm.

### 3.5 Collagen VI deposition and turnover

**Fig. 5:**
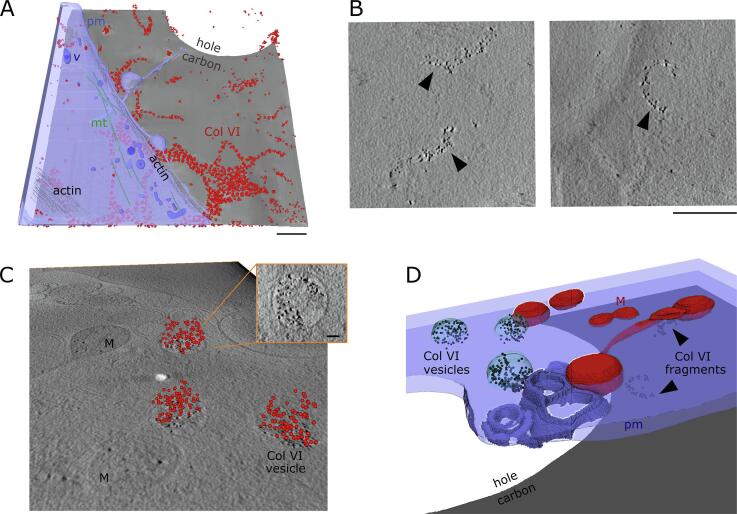
CSTET imaging of collagen VI degradation and uptake. Labeled features are plasma membrane (pm), collagen VI (Col VI), vesicles (v), mitochondria (M), microtubules (mt), and actin. (A) Tomogram segmentation (right) showing extensive collagen VI networks coating the carbon in the vicinity of a cell. Gold beads labeling collagen VI are shown as red spheres in the segmentation. Scale bar is 500 nm. (B) Gold-labeled collagen VI fragments under a cell, indicated by arrowheads. Scale bar is 500 nm. (C) A section of a tomogram is shown at an angle to emphasize the three-dimensionality of the gold-particle organization (red spheres) within intracellular vesicles. Inset shows a slice through a gold-filled vesicle. Scale bar in inset is 100 nm. (D) Segmentation of the organelles and plasma membrane in the region corresponding to panel C, rotated from that view by 180° on an axis perpendicular to the carbon. (For interpretation of the references to colour in this figure legend, the reader is referred to the web version of this article.)

The ability to visualize cells and matrix in a near-native state using CSTET provided insights into collagen VI deposition and turnover by cells. As noted above, collagen VI was dispersed to the cell surroundings upon secretion, and it adhered and assembled into networks and filaments on the carbon support. Collagen VI labeling could be seen extensively in areas lacking cells and to some extent under cells near cell edges (Fig. 5A). It is likely that thorough labeling of collagen VI between cells and the carbon support was prevented by the inability of the antibody to diffuse into these regions. Therefore, the limited labeling that was detected under cells implies that either the cells migrated onto the labeled collagen VI or the adhesion of these cell regions to the support was loose and fluctuating, allowing antibody penetration. In a sample that was exposed to primary antibody labeling for about four hours, longer than our typical protocol, collagen VI was again detected under cells to some extent, but in this case the networks appeared fragmented (Fig. 5B). Though it is possible that fibrils coated with antibody, as in this experiment, are processed by the cell differently from unlabeled collagen VI, insights into collagen VI turnover can be gained from this observation. Collagen VI fragments were about 700 nm in length and consisted of about 50 gold beads and 8 globular collagen VI regions (Fig. 5B). In addition, three large intracellular vesicles (∼400 nm in diameter) containing gold beads, ranging from 100 to 160 beads per vesicle, were observed (Fig. 5C). The contents of the vesicle may thus correspond to two or three collagen VI fragments of the kind detected extracellularly. Due to the diameter of the vesicles being shorter than the fragment lengths, the fragments would not remain extended once internalized. The gold beads in the vesicle closest to the cell periphery were seen proximal to the vesicle membrane in a dome-like arrangement (Fig. 5C, inset), with the length of the gold-bead arch corresponding to the length of typical extracellular collagen VI fragments. We speculate that the vesiculation process is initiated by binding of cell-surface receptors to collagen VI fragments, which curve accordingly as the vesicle forms. The gold beads in the other two vesicles were more evenly distributed in the vesicle lumen, suggesting that the fragments detach from the edge as the vesicles leave the plasma membrane. Uptake of exogenously supplied collagens by fibroblasts has been measured (Engelholm et al., 2003, Everts et al., 1985, Everts and Beertsen, 1992, Kjøller et al., 2004), but the sizes of collagen VI fragments, their spatial relation to the cell, and their quantity, density, and distribution in intracellular vesicles have not, to our knowledge, been recorded previously.

## 4 Discussion

In this work we imaged labeled ECM components in three dimensions, in the context of fully vitrified cultured primary fibroblast cells that were not chemically fixed nor stained. The two prevalent ECM proteins of interest, fibronectin and collagen VI, showed different modes of deposition in relation to cells and to the supporting carbon substrate. Fibronectin tended to cover cells in a branching and merging pattern running parallel to the long cell axes. Collagen VI was deposited extensively on the carbon support in regions not covered by cells, as well as on cells and to some extent under cells (*i.e*., between the cells and the carbon support). Additional unobserved collagen VI may have been present under cells where it was inaccessible to antibody labeling. The large amount of collagen VI detected in open lattice-like networks on the opposite side of the carbon layer from the cells suggests that secreted collagen VI is not tightly retained by the cells that produce it, but rather is distributed to and assembles within the local environment. This observation is consistent with the role of interstitial fibroblasts in providing collagen VI to muscle cells (Bönnemann, 2011).

A number of conclusions can be drawn regarding the structure and organization of collagen VI *in situ* from the CSTET measurements. We observe that collagen VI fibrils in the context of cell cultures are flexible, such that fibrils are often gently curved. In certain cases, curvature is more pronounced, such that a segment containing 6 globular beads along an individual fibril is seen to arc through about 120° (Fig. 4B). Sharper angles may represent other types of junction in the network, such as those responsible for node formation. Collagen VI appears to have a lesser tendency than fibronectin for lateral interactions along filaments, though large, loose bundles of collagen VI were often observed.

A major finding from the quantitative analysis of collagen VI in intact fibroblast cultures is the repeat distance between globular beads in the microfibrils. The distance we observe, approximately 85 nm, is shorter than the typical values around 100 nm reported in other studies (Baldock et al., 2003, Bruns, 1984, Engel et al., 1985, Engvall et al., 1986). The triple-helical region of collagen VI is composed of about 335 amino acids per strand, expected to span about 95 nm using a value of 0.87 nm for every amino acid triplet, as derived from crystal structures of collagen triple helix peptides. However, higher-order collagen VI supramolecular assembly may involve supercoiling of the triple helices (Knupp and Squire, 2001), which would shorten the distance spanned by each triple helix along the fibril axis. Notably, collagen triple helices have been reported to melt and stretch (Leikina et al., 2002, Makareeva et al., 2008). In rotary shadowing electron micrographs of collagen VI that had been fixed and dehydrated, the inter-bead fibers were seen to have separated from one another, indicating disruption of supercoiling (Engvall et al., 1986, Furthmayr et al., 1983, Martoni et al., 2013, Zhang et al., 2002). It is known, moreover, that “combing” of collagen VI microfibrils lengthens the repeat distance (Baldock et al., 2003). In addition to being affected by the degree of supercoiling, the inter-bead distance depends also on the size and organization of the globular bead region itself (Godwin et al., 2017), which has been observed to change in response to force (Baldock et al., 2003). Though a structural justification of the 85 nm repeat distance we observe is not possible at this point in time, we find this period to be a highly reproducible phenomenon of gold-labeled collagen VI in cryo-preserved fibroblast cultures. Our observations are biased toward collagen VI in association with near-confluent cells or deposited passively on carbon in hydrated conditions. It will be interesting to determine whether collagen VI subjected to forces exerted by cell protrusion or retraction might display different repeat distances.

In addition to new perspectives on the organization of collagen VI fibrils and networks in a rich ECM environment, observations were made regarding the assembly and remodeling of collagen VI and its interactions with cells. In particular, collagen VI was seen draped over cell-membrane protrusions. Collagen VI in these locations was found as thick bundles, in contrast to the flat, two-dimensional polygonal networks assumed by collagen VI deposited on the carbon of the grids. This finding may indicate reorganization of collagen VI by cells penetrating under the lattice. Fibroblasts rearrange ECM fibrils during migration or during cyclic membrane retraction and extension (Grinnell and Petroll, 2010, Gudzenko and Franz, 2015, Gudzenko and Franz, 2013). The thick bundles of collagen VI with the cell protrusions underneath them as shown in Fig. 4E appear to present a snapshot of cell edge dynamics in the process of rearranging the polygonal collagen VI network into bulkier three-dimensional bundles. Fibroblasts have been shown to rearrange another collagen type, collagen I, by invading nanoscale regions underneath the fibers (Gudzenko and Franz, 2013), and cells may remodel collagen VI in a similar manner.

An important topic in cell biology is how cells are affected by mechanical forces and by the rigidity of the microenvironment (Discher et al., 2005, Vogel and Sheetz, 2006). CSTET analysis of cells and ECM in contact with the holey carbon layer on which the cells were grown illuminated morphological responses to variations in the stiffness of the cell support. For example, we often observed bleb formation at holes in the carbon (Fig. 3C, D and Fig. 5D). In these regions, the plasma membrane detached from cortical actin, and the membrane and cytosol flowed into the space with lower mechanical resistance, as occurs for cell blebbing in other contexts (Collier et al., 2017). Cytoskeletal elements and fibronectin appeared relatively unperturbed by this phenomenon, but intracellular organelles, such as mitochondria, were visibly affected. In the thick, three-dimensional volume offered by CSTET visualization, the striking behavior of a mitochondrion sending an offshoot under a broad bundle of parallel actin filaments and into the bleb was observed (Fig. 3D). The constriction of the mitochondrion under the actin bundle is indicative of an impending fission event. Mitochondrial fission in response to a discontinuity in the physical structure of the microenvironment as imaged here using CSTET corresponds to the observation made using fluorescence microscopy that mitochondria undergo fission when subjected to mechanical forces or at the edges of grooves in a cell-culture substrate (Helle et al., 2017). In the CSTET tomogram, the bleb and its mitochondrial offshoot appear to have pooled in a pocket delineated by fibronectin bundles crossing the hole. Importantly, the thick bundles of actin extending along the long edges of the fibroblasts were insufficient to prevent the flow of cytosol into cavities in the microenvironment, but the extracellular presence of fibronectin, likely together with other, unlabeled ECM components, kept cell blebbing in check. The simultaneous visualization of ECM and intracellular organelles shows how diverse subcellular components respond to mechanical irregularities, which are likely to be a common feature of the complex three-dimensional arrangements of cells and ECM within tissues and organs.

The CSTET method (Wolf et al., 2014) provides low nanometer resolution in the lateral dimensions, with slightly poorer resolution in the z direction due to the missing wedge effect, yielding ultrastructure maps for regions spanning a few microns in width and up to one micron in thickness. Organelles and other intracellular constituents are visualized by natural contrast arising from differences in elemental composition and density (Rez et al., 2016, Wolf et al., 2017, Wolf et al., 2015, Wolf et al., 2014). Extracellular components such as individual ECM fibrils may be mapped by immunogold labeling as done here. Unlike immunogold labeling of isolated ECM components in solution, which has been suggested to induce fibril aggregation (Kielty et al., 1991), labeling in the context of the native, highly crosslinked three-dimensional ECM network (Fig. 1) is likely to preserve pre-existing ultrastructure. Though lacking the resolution of TEM analysis of purified components, the CSTET approach enables the quantitative investigation of multi-scale assembly modes *in situ*. Furthermore, by providing information on numerous unlabeled cellular components together with labeled ECM fibrils, CSTET enables unbiased, exploratory research on cell-ECM interactions. In addition, the high contrast of gold in STEM mode allows for precise localization of extremely small fiducials (6 nm in our study), even within thick regions of the cell.

In this manner, CSTET complements other structural techniques for analysis of ECM organization and associations. Super-resolution fluorescence microscopy has provided details on fibronectin organization in cell cultures and tissues (Früh et al., 2015), but this methodology is limited to the investigation of those macromolecules that are specifically labeled. A cryo-TEM analysis revealed the collagen VI bead structure to about 5 nm resolution (Godwin et al., 2017), and additional insights into the structures of ECM components are likely to be provided in the future, enabled by improved cryo-TEM technologies. Such structural studies, however, typically require removal of ECM components from their natural context, or, more commonly, recombinant production of limited ECM fragments in isolation. SEM in turn provides topographic images of ECM in intact cell cultures (Fig. 1) and tissues (Gillies et al., 2017, Keene et al., 1988), as well as in reconstituted form (Miron-Mendoza et al., 2010), but under dehydrated conditions. AFM has provided images of ECM microfibrils (Baldock et al., 2003, Godwin et al., 2017, Gudzenko and Franz, 2015, Gudzenko and Franz, 2013, Prein et al., 2016), and of ECM on the basal side of fixed fibroblasts (Gudzenko and Franz, 2013). CSTET has the advantage of imaging thicker regions of the cell (*e.g*., 600 nm depth shown in Fig. 4D) than would be viewed using conventional heavy metal-stained thin-section TEM or cryo-TEM (Villa et al., 2013), while preserving the surrounding ECM in its entirety. Imaging of thick cell regions provides information on how ECM is distributed with respect to cell contours in a non-perturbing manner.

Cell-ECM interactions on the nanoscale may impact cell morphology on the micron scale (Gudzenko and Franz, 2013), in turn affecting organellar and cellular function. In this study, we observed how fibronectin bundles span micron-scale cavities in the cell microenvironment, represented in our experiment by holes in the solid carbon layer of the grid, and buttress cell blebs that contain organelles flowing from the main cell body. We also observed how collagen VI is disseminated broadly from secreting fibroblasts, assembles in their vicinity into semi-regular open networks, and is then bundled into thick filaments or taken back up into cells in fragments. The tomographic data presented here provide a new perspective on the role of fibronectin in cell support and orientation, and on the formation of an extended collagen VI network for cell locomotion and intercellular communication. We anticipate that additional discoveries relating ECM structure and composition to the arrangement and behavior of intracellular organelles will be facilitated by CSTET visualization.

## Declaration of Competing Interest

The authors declare that they have no known competing financial interests or personal relationships that could have appeared to influence the work reported in this paper.

## Appendix A Supplementary data

Supplementary Movie S1 Orthoslices and segmentation of a fibronectin labeled tomogram, corresponding to Fig. 3D.

Supplementary Movie S2 Orthoslices and segmentation of a collagen VI labeled tomogram, corresponding to Fig. 4F.
